# Flashback

**DOI:** 10.36834/cmej.69849

**Published:** 2020-03-16

**Authors:** Rebecca Zhao

**Affiliations:** 1 University of Saskatchewan, Saskatchewan, Canada

**Figure:**
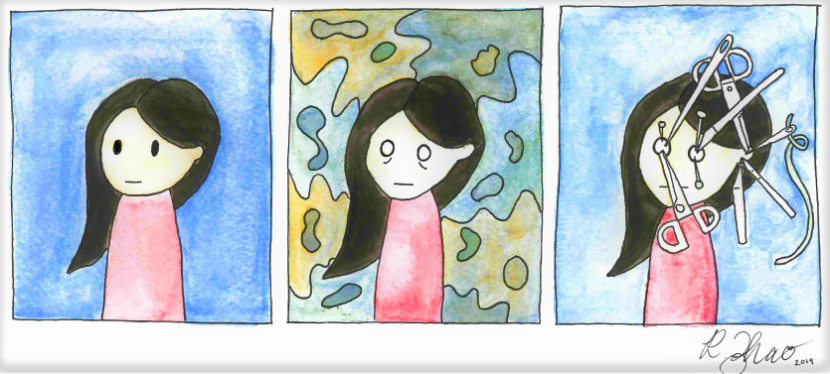


Inspired by graphic health narratives—or comics as some scholars may define them—my painting “Flashback” shares my experience of recalling a traumatic incident. My aim for this painting was to evoke a visceral reaction of discomfort (particularly with the sharp objects in the character’s eyes) so that viewers may feel some semblance of what I had felt. Flashback was exhibited at the Surgical Humanities Day at the University of Saskatchewan in September 2019.

